# Supplementary material for: A fault-intrusion-tolerant system and deadline-aware algorithm for scheduling scientific workflow in the cloud
Source: PeerJ Comput Sci. 2021 Nov 2;7:e747. doi: 10.7717/peerj-cs.747 (PMC8576554; doi:10.7717/peerj-cs.747)
Supplement: Supplemental Information 6 [file peerj-cs-07-747-s006.zip › Myproject1.1/docs/org/workflowsim/clustering/class-use/TaskSet.html]

Uses of Class org.workflowsim.clustering.TaskSet


JavaScript is disabled on your browser.


- Overview
- Package
- Class
- Use
- Tree
- Deprecated
- Index
- Help

- Prev
- Next

- Frames
- No Frames

- All Classes

## Uses of Class org.workflowsim.clustering.TaskSet

- Packages that use TaskSet

  | Package | Description |
  | --- | --- |
  |  |  |
  | --- | --- |
  | org.workflowsim.clustering |  |
  | org.workflowsim.clustering.balancing |  |
  | org.workflowsim.clustering.balancing.methods |  |
  | org.workflowsim.clustering.balancing.metrics |  |
- - ### Uses of TaskSet in org.workflowsim.clustering

    Methods in org.workflowsim.clustering that return types with arguments of type TaskSet

    | Modifier and Type | Method and Description |
    | --- | --- |
    |  |  |
    | --- | --- |
    | `java.util.ArrayList<TaskSet>` | TaskSet.`getChildList()` Gets the child list |
    | `java.util.ArrayList<TaskSet>` | TaskSet.`getParentList()` Gets the parent list |
  - ### Uses of TaskSet in org.workflowsim.clustering.balancing

    Methods in org.workflowsim.clustering.balancing that return types with arguments of type TaskSet

    | Modifier and Type | Method and Description |
    | --- | --- |
    |  |  |
    | --- | --- |
    | `java.util.Map<java.lang.Integer,java.util.ArrayList<TaskSet>>` | BalancedClustering.`getCurrentTaskSetAtLevels()` Gets the current tasks per level |
  - ### Uses of TaskSet in org.workflowsim.clustering.balancing.methods

    Methods in org.workflowsim.clustering.balancing.methods that return TaskSet

    | Modifier and Type | Method and Description |
    | --- | --- |
    |  |  |
    | --- | --- |
    | `protected TaskSet` | HorizontalDistanceBalancing.`getCandidateTastSet(java.util.ArrayList<TaskSet> taskList, TaskSet checkSet, int clusters_size)` Gets the potential candidate taskSets to merge |
    | `protected TaskSet` | HorizontalImpactBalancing.`getCandidateTastSet(java.util.ArrayList<TaskSet> taskList, TaskSet checkSet, int clusters_size)` Gets the potential candidate taskSets to merge |

    Methods in org.workflowsim.clustering.balancing.methods with parameters of type TaskSet

    | Modifier and Type | Method and Description |
    | --- | --- |
    |  |  |
    | --- | --- |
    | `void` | BalancingMethod.`addTaskSet2TaskSet(TaskSet tail, TaskSet head)` Add all the tasks in tail to head and then clean tail. |
    | `protected TaskSet` | HorizontalDistanceBalancing.`getCandidateTastSet(java.util.ArrayList<TaskSet> taskList, TaskSet checkSet, int clusters_size)` Gets the potential candidate taskSets to merge |
    | `protected TaskSet` | HorizontalImpactBalancing.`getCandidateTastSet(java.util.ArrayList<TaskSet> taskList, TaskSet checkSet, int clusters_size)` Gets the potential candidate taskSets to merge |

    Method parameters in org.workflowsim.clustering.balancing.methods with type arguments of type TaskSet

    | Modifier and Type | Method and Description |
    | --- | --- |
    |  |  |
    | --- | --- |
    | `protected TaskSet` | HorizontalDistanceBalancing.`getCandidateTastSet(java.util.ArrayList<TaskSet> taskList, TaskSet checkSet, int clusters_size)` Gets the potential candidate taskSets to merge |
    | `protected TaskSet` | HorizontalImpactBalancing.`getCandidateTastSet(java.util.ArrayList<TaskSet> taskList, TaskSet checkSet, int clusters_size)` Gets the potential candidate taskSets to merge |
    | `void` | HorizontalDistanceBalancing.`process(java.util.ArrayList<TaskSet> taskList)` Sort taskSet based on their impact factors and then merge similar taskSet together |
    | `void` | HorizontalImpactBalancing.`process(java.util.ArrayList<TaskSet> taskList)` Sort taskSet based on their impact factors and then merge similar taskSet together |
  - ### Uses of TaskSet in org.workflowsim.clustering.balancing.metrics

    Method parameters in org.workflowsim.clustering.balancing.metrics with type arguments of type TaskSet

    | Modifier and Type | Method and Description |
    | --- | --- |
    |  |  |
    | --- | --- |
    | `double` | BalancingMetric.`getMetric(java.util.ArrayList<TaskSet> list)` Gets the metric value. |
    | `double` | DistanceVariance.`getMetric(java.util.ArrayList<TaskSet> list)` Returns the distance variance of a list of taskSets |
    | `double` | HorizontalRuntimeVariance.`getMetric(java.util.ArrayList<TaskSet> list)` Returns the standard deviation of runtime |
    | `double` | ImpactFactorVariance.`getMetric(java.util.ArrayList<TaskSet> list)` Returns the standard deviation of their impact factors |
    | `double` | PipelineRuntimeVariance.`getMetric(java.util.ArrayList<TaskSet> list)` Returns the standard deviation of the pipeline runtime |

- Overview
- Package
- Class
- Use
- Tree
- Deprecated
- Index
- Help

- Prev
- Next

- Frames
- No Frames

- All Classes
